# Quercetin protects PC-12 cells against hypoxia injury by down-regulation of miR-122

**DOI:** 10.22038/ijbms.2019.30224.7287

**Published:** 2019-04

**Authors:** Rui Yan, Huiling Tian, Zhongxiu Du

**Affiliations:** 1 Department of Children Rehabilitation, Women & Children’s Health Care Hospital of Linyi, Linyi 276016, Shandong, China

**Keywords:** AMPK pathway, Hypoxia injury, miR-122, Quercetin, Wnt/β-catenin pathway

## Abstract

**Objective(s):**

Impairment of nerve cells of brain induced by hypoxia results in energy-deprivation and dysfunction, which accompanies with neurons apoptosis. Improving function of nerve cells is important for treating cerebral anoxia. This study aimed to investigate the role of Quercetin (Quer) in hypoxia-induced injury of pheochromocytoma (PC-12) cells.

**Materials and Methods:**

PC-12 cells were cultured under anoxic condition for induction of hypoxia injury and/or treatment with Quer, transfection with pre-miR-122, anti-miR-122 or their negative controls. After Quer treatment, viability, migration, and cell apoptosis of PC-12 cells were analyzed by CCK-8 assay, transwell assay and flowcytometry analysis, respectively. Cell proliferation-associated proteins and cell apoptosis-associated proteins were analyzed by Western blot. Relative miR-122 expression in Quer-treated cells or transfection efficacy of miR-122 was analyzed by qRT-PCR. Finally, main components in AMP-activated protein kinase (AMPK) and Wnt/β-catenin signaling pathways were analyzed by Western blot.

**Results:**

Quer alleviated hypoxia-induced injury in PC-12 cells by increasing viability, promoting cell proliferation, enhancing migration and repressing apoptosis. Also, miR-122 was down-regulated in Quer-treated cells. miR-122 overexpression decreased cell viability and migration, and increased cell apoptosis in hypoxia- treated PC-12 cells, but miR-122 silencing led to the opposite results. We also found that AMPK and Wnt/β-catenin signaling pathways were activated by Quer in hypoxia-induced injury, but were inactivated in hypoxia-induced cells by overexpression of miR-122.

**Conclusion:**

Quer could repress hypoxia-induced injury in PC-12 cells by activating AMPK and Wnt/β-catenin signaling pathways via down-regulation of miR-122.

## Introduction

Neonatal asphyxia causes various diseases such as hypoxic–ischemic encephalopathy (HIE) and leads to death in almost one quarter of the 3.6 million deaths (1). In addition, two phases of injury occur of which the first phase is an immediate phase of neuronal cell injury and exhaustion of energy stores, which is followed by a secondary phase largely mediated by oxidative stress, inflammatory cytokines and apoptosis (2). Unfortunately, ill newborns and infants suffer from high risk of these events, which is mainly resulted from perinatal asphyxia in neonatal periods during neonatal respiratory or cardiovascular failure and severe operation (3). Some children die of treatment failure and the survivors often suffer from mental retardation, cerebral palsy and other sequel (4). Although there has been great progress in neonatal nursing, management strategy that minimizes the incidence of hypoxic encephalopathy has been limited (5). However, high and growing incidence have consistently displayed in this disappointing scenario. Therefore, it is important to find adjuvant therapies that can be used for asphyxia-related diseases in neonates. 

Some natural compounds extracted from plants play an important role in improvement of brain function and hemodynamic instability after severe hypoxia, such as ligustilide (6), Tanshinone IIA (7) and Quercetin (Quer) (8). Quer (3, 5, 7, 30, 40-pentahydroxyflavone) belongs to a broad group of polyphenolic flavonoid substances presenting in beverages, food, plants and many other sources. Quer processes a lot of biological and pharmacological activities. Beyond the treatment of cerebral ischemia (8, 9), extensive beneficial properties of Quer have been reported, involving anti-inflammatory diseases (10), anti-tumor and regulation of tumor-associated multidrug resistance (11), anti-atherosclerotic (12) and anti-hypertensive effects (13), as well as anti-diabetic and antioxidant efficacy (14).

The neuroprotective effect of Quer has been widely reported (15). Quer was reported to alleviate traumatic brain injury through adaptation of mitochondrial function via mediation of peroxisome proliferator-activated receptor gamma coactivator 1-alpha (PGC-1α) pathway (16). In hypoxia-induced neuronal injury, Quer was found to be an inhibitor of μ-calpain, which is a calcium ion-activated intracellular cysteine protease, and can cause serious cellular insult, resulting in cell death in diverse pathological conditions (17). Quer improved brain function and maintained hemodynamic stability in newborn piglets submitted to the severe hypoxic or hypoxic/ischemic episode (8). Additionally, Quer meliorated hypoxia-induced cognitive deficits by promoting remyelination in neonatal rat (18). Therefore, Quer has been shown to be an effective compound to repress hypoxia-induced brain damage.

MicroRNAs (miRNAs) that referred to endogenous non-coding RNAs of 19–24 nucleotides in length, affect various functions by interfering with the translation or stability of target mRNA (19). Among all these identified miRNAs, microRNA-122 (miR-122) has important functions including essential metabolic, anti-inflammatory, and anti-tumorigenic effects (20). In addition, miR-122 was found to be up-regulated by Quer in dietary supplements with anti-inflammatory functions (21). Furthermore, miR-122 regulates hypoxia-inducible factor-1 and vimentin (22). Therefore, we hypothesized that miR-122 might also play an important role in effect of Quer on hypoxia-treated pheochromocytoma (PC-12) cells. In this study, the effect of Quer was investigated on PC-12 cells exposed to hypoxia treatment and the underlying mechanism was also explored. 

## Materials and Methods

***Cell culture and treatment***

Rat PC-12 cells provided by Kunming Institute of Zoology (Kunming, China) were used throughout this study. Cells were seeded into flasks (1×10^4^ cells/ml) in Dulbecco’s modified Eagle medium (DMEM) supplemented with fetal bovine serum (10% (v/v)), penicillin (100 U/ml), and streptomycin (100 μg/ml). Cells were incubated at 37 ^°^C under humid conditions containing 5% CO_2_. O_2_ concentration with 3% and 21% were respectively used as the hypoxia and normoxia culture conditions. PC-12 cells were cultured for 24 hrs under hypoxic conditions. Quer (Q4951, ≥ 95% (HPLC), solid, Sigma-Aldrich, St. Louis, MO, USA) was dissolved in dimethyl sulfoxide (DMSO) in 100 mM. Cells were treated by Quer at different concentrations ranging from 10 μM to 40 μM for 24 hrs. 

**Figure 1:**
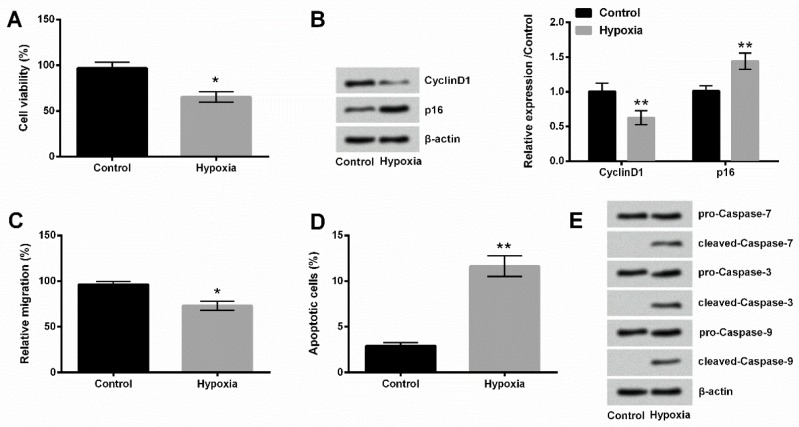
Hypoxia induced PC-12 cell injury. (A) Viability of oxygen-starved cells was inhibited. (B) Cyclin D1 was down-regulated and p16 was up-regulated in oxygen-starved PC-12 cells. (C) Migration of oxygen-starved PC-12 cells was inhibited. (D) Apoptosis of oxygen-starved PC-12 cells was increased. (E) The activated forms of caspase-7/3/9 proteins were detected in PC-12 cells after hypoxia injury

**Figure 2.**
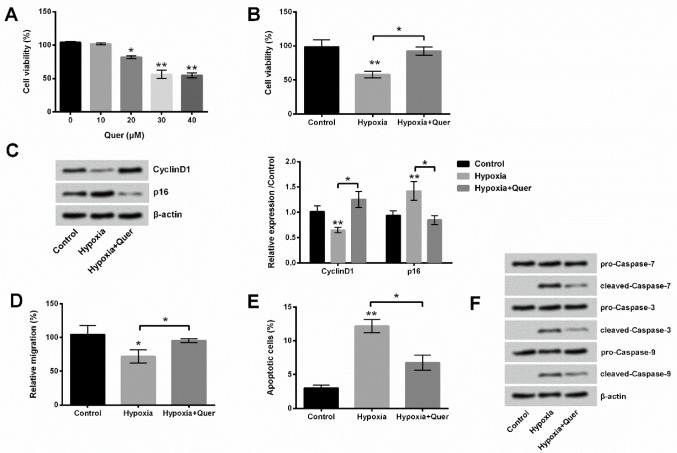
Quercetin (Quer) ameliorated hypoxia-induced PC-12 cell injury. (A) Quer with concentration of 10 μM showed no toxic effect on viability. (B) Quer treatment increased PC-12 cell viability. (C) Quer increased Cyclin D1 expression and decreased p16 expression. (D) Quer treatment promoted migration of PC-12 cells. (E) Quer treatment repressed apoptosis of PC-12 cells. (F) Quer decreased cleaved-caspase-7/3/9 expressions in PC-12 cells

**Figure 3:**
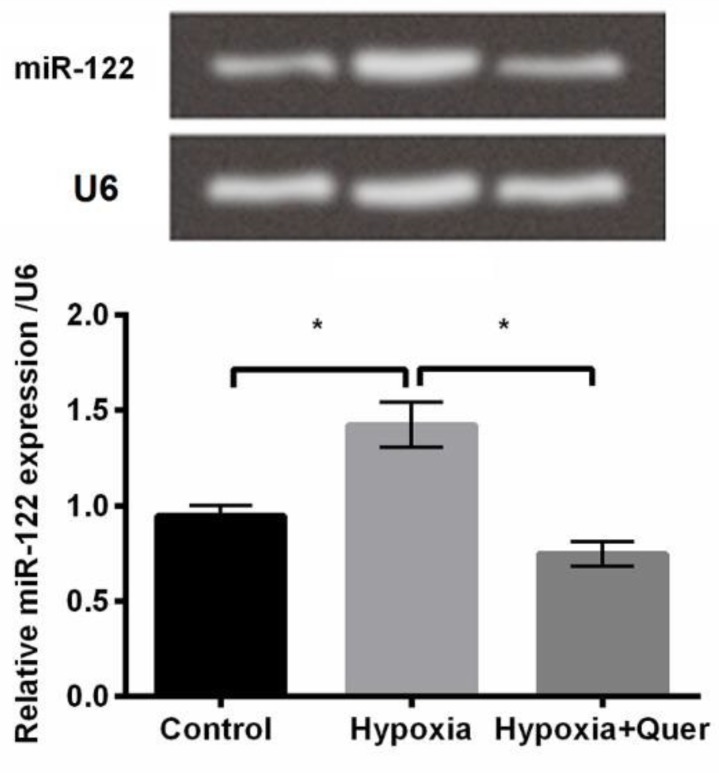
Relative expression of miR-122 in PC-12 cells was increased after hypoxia injury and then repressed after Quercetin treatment

**Figure 4:**
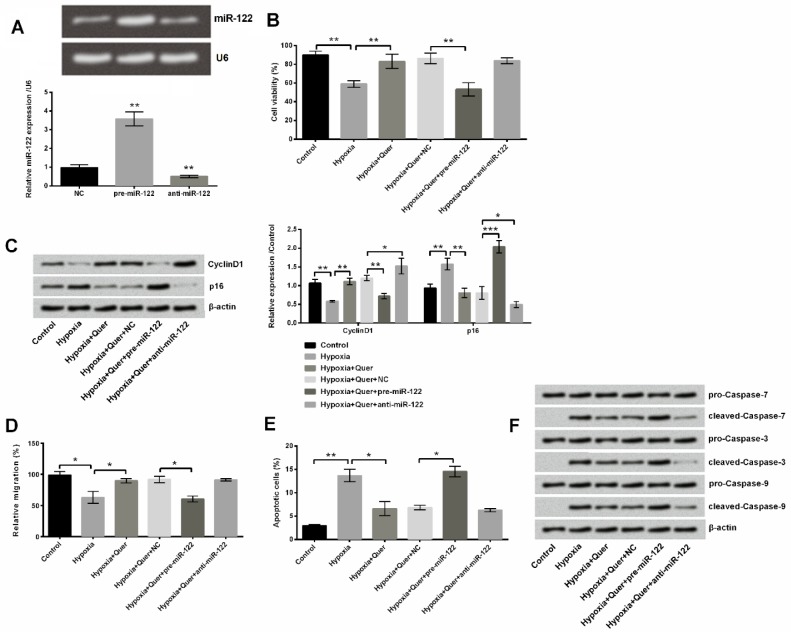
Up-regulating miR-122 enhanced hypoxia-induced injury, and down-regulating miR-122 protected PC-12 cells. (A) miR-122 expression was altered after transfection with pre-miR-122 and anti-miR-122. (B) miR-122 overexpression decreased viability. (C) miR-122 overexpression decreased Cyclin D1 expression and increased p16 expression, but silence of miR-122 showed the contrary effect. (D) miR-122 overexpression decreased migration. (E) miR-122 overexpression increased apoptosis. (F) miR-122 overexpression increased expressions of cleaved-caspase-7/3/9, but silence of miR-122 decreased their expressions

**Figure 5:**
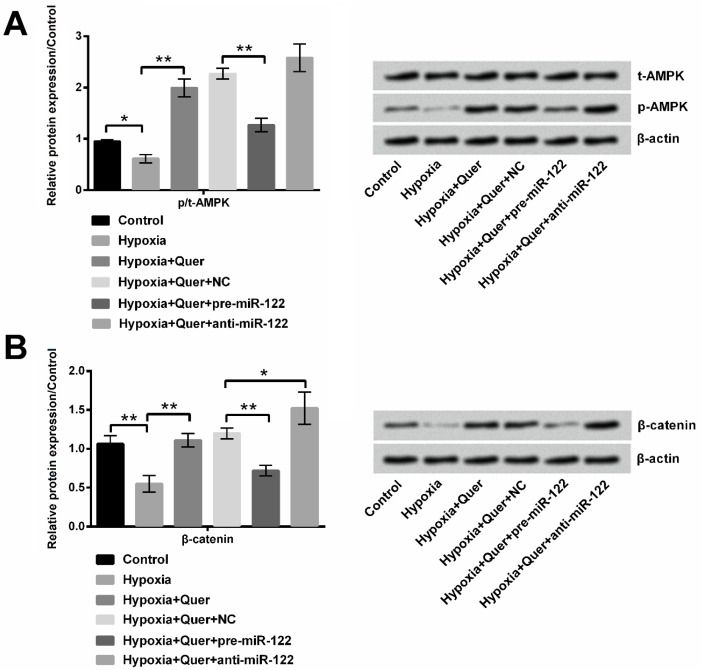
Quercetin (Quer) protected PC-12 cells through activating Adenosine 5‘-monophosphate (AMP)-activated protein kinase (AMPK) and Wnt/β-catenin signaling pathways. (A) miR-122 regulated the effect of Quer on activation of AMPK signaling pathway. (B) miR-122 regulated the effect of Quer on activation of Wnt/β-catenin signaling pathway

***CCK-8 assay***

Cell suspension was inoculated in a 96-well plate (100 μl/well). Cells were pre-incubated in the plate in a humidified incubator (at 37 ^°^C, 5% CO_2_). 10 μl of the Cell Counting Kit-8 (CCK-8) solution was added to each well of the plate carefully avoiding introducing bubbles to the wells since they interfere with the optical density (OD) reading. The plate was incubated for 2 hrs in the incubator and the absorbance at 450 nm was measured using a microplate reader (Bio-Rad, Hercules, CA) after incubation.

***qRT-PCR analysis***

Total RNA was isolated using TRIzol reagent (Invitrogen, Carlsbad, CA, USA). First strand cDNA was reversely transcribed from 1 μg of total RNA using reverse transcriptase and random hexamers from RevertAid^TM^ First Strand cDNA Synthesis Kit (Fermentas, Burlington, Ontario, Canada). PCR was performed with rTaq (TaKaRa, Tokyo, Japan) in a DNA thermal cycler (Maxygen, Sunnyvale, CA, USA). The expressions of mRNA were normalized to β-actin expression. For detecting miR-122 expression, Taqman MicroRNA Reverse Transcription Kit and Taqman Universal Master Mix II (Applied Biosystems, Foster City, CA, USA) using probe for miR-122 were used to synthesize cDNA and perform PCR. Its expression was normalized to U6 expression. The relative expressions of miR-122 and tested mRNAs were calculated using 2^−ΔΔCt^ method.

***miRNA transfection***

Pre-miR-122, anti-miR-122 and their negative control (NC) were synthesized by GenePharma Co. (Shanghai, China) and transfected into PC-12 cells using Lipofectamine 2000 reagent (Invitrogen) in accordance with the manufacturer’s use instructions.

***Apoptosis assay***

Cells were washed in phosphate-buffered saline (PBS) by gently shaking or pipetting up and down and then were resuspended in 195 μl binding buffer (1×) at a density of 5×10^5^ cells/ml. Afterwards, 5 μl Annexin V-fluorescein isothiocyanate (FITC) solution (Sangon Biotech, Shanghai, China) was added into cell suspension and the obtained mixture was incubated for 10~15 mins at room temperature protected from light. Cells were then washed with 200 μl binding buffer (1×) and resuspended in 190 μl binding buffer (1×) after centrifugation. After added with 10 μl propidium iodide (PI, Sangon Biotech), cells were analyzed by flow cytometry (Beckman Coulter, Fullerton, CA, USA) within 4 hr.

***Migration assay***

Transwell chamber with pore size of 8 μm was used for detecting migratory capacity of PC-12 cells. 200 μl of serum-free medium and 600 μl of complete medium were added in the upper chamber and lower chamber, respectively. PC-12 cells were seeded in the upper chamber. After incubation at 37 ^°^C, migratory cells on the lower side of the filter were fixed with methanol and also stained with crystal violet for counting. 

***Western blot***

The protein was extracted from PC-12 cells using RIPA lysis buffer (Beyotime Biotechnology, Shanghai, China) and subsequently quantified by BCA™ Protein Assay Kit (Pierce, Appleton, WI, USA). Protein was resolved over 12% SDS-PAGE and transferred to the PVDF membrane. After blocking in 5% non-fat milk, membranes were orderly incubated with primary antibodies (4 ^°^C, overnight) and secondary antibodies marked by horseradish peroxidase (room temperature, 1 hr). The primary antibodies were used as follows: Cyclin D1 (ab134175), p16 (ab51243), pro caspase-7 (ab69540), cleaved caspase-7 (ab2323), pro caspase-3 (ab44976), cleaved caspase-3 (ab49822), pro caspase-9 (ab2013), cleaved caspase-9 (ab52298), AMP-activated protein kinase (AMPK) (ab131512), phosphorylated AMPK (p-AMPK) (ab23875), β-catenin (ab32572), and β-actin (ab8227) were purchased from Abcam (Cambridge, MA, USA). Signals were captured by enhanced chemiluminescence (ECL) method and quantitative band-intensity analysis was performed using Image Lab™ Software (Bio-Rad, Shanghai, China).

***Statistical analysis***

Data were presented as mean±standard deviation (SD). The one-way analysis of variance (ANOVA) with the Bonferroni multiple comparisons *post-* *hoc *test was used to perform statistical analysis using Graphpad Prism version 6.0 software (Graph Pad Software, San Diego California, USA). In all graphs, *P* value<0.05 was considered as statistically significant between control and treated groups. 

## Results

***Hypoxia treatment induced PC-12 cell injury***

PC-12 cells, starved for oxygen, showed significantly decreased cell viability according to CCK-8 assay (*P*<0.05, Figure 1A), significantly reduced Cyclin D1 expression (*P*<0.01) and enhanced p16 expression (*P*<0.01, Figure 1B) according to Western blot analysis. PC-12 cells also exhibited significantly decreased migrated capacity (*P*< 0.05, Figure 1C) based on analysis of transwell chamber assay. The effect of hypoxia on apoptosis of PC-12 cells was also analyzed by flow cytometry and Western blot. Results showed that apoptotic cell rate was significantly increased (*P*<0.01, Figure 1D), and caspase-7, caspase-3, and caspase-9 were all activated (Figure 1E). 

***Quer alleviated hypoxia-induced PC-12 cell injury***

The effects of different concentrations of Quer on viability of PC-12 cells were firstly analyzed to choose the maximum non-toxic dosage of Quer. Figure 2A showed that Quer at concentrations of 20, 30, and 40 μM obviously decreased cell viability (*P*<0.05 or *P*<0.01). Therefore, a dose of 10 μM was used for subsequent studies. Quer attenuated the effects of hypoxia treatment on cell viability, proliferation-associated factors, migration, and apoptosis. In detail, Quer significantly improved cell viability (*P*<0.05, Figure 2B), up-regulated Cyclin D1 expression (*P*<0.05), diminished p16 expression (*P*<0.05, Figure 2C), enhanced migration (*P*<0.05, Figure 2D), decreased apoptotic cell rate (*P*< 0.05, Figure 2E), and reduced expressions of cleaved-caspase-7/3/9 (Figure 2F). 

***Quer down-regulated miR-122 expression***

The underlying action mechanism of Quer in protective function on PC-12 cells with oxygen-deprivation was investigated. It was found that miR-122 expression was up-regulated in hypoxia-injured cells relative to control group (*P*<0.05, Figure 3); however, its expression was decreased after Quer treatment compared to hypoxia treatment (*P*<0.05, Figure 3). Then, we speculated that miR-122 might be involved in the function of Quer and thereby, we analyzed the effects of altered expression of miR-122 in PC-12 cells. 

***Quer protected PC-12 cells against hypoxia injury through down-regulating miR-122***

The expression of miR-122 was altered by transfection assay, with a significant increase in pre-miR-122 treatment (*P*<0.01) and a significant decrease in anti-miR-122 treatment (*P*<0.01, Figure 4A). Next, miR-122 overexpression was observed to impair the viability-promoting effect of Quer in hypoxia-induced PC-12 cells compared to NC (*P*<0.01), but miR-122 silence had no significant effect on viability (Figure 4B). In addition, under Quer treatment, miR-122 overexpression decreased Cyclin D1 expression but increased p16 expression, whereas repression of miR-122 exhibited the contrary effects on their expressions in hypoxia-induced PC-12 cells compared to NC (*P*<0.05, *P*<0.01 or *P*<0.001, Figure 4C). Transfecting pre-miR-122 and treatment with Quer significantly decreased migratory ability of hypoxia-induced PC-12 cells compared to NC (*P*<0.05, Figure 4D). Finally, the effect of miR-122 on apoptosis of hypoxia-induced PC-12 cells was analyzed. Cell apoptotic rate was increased after elevating miR-122 expression (*P*<0.05) but no significant change occurred after suppressing miR-122 by the treatment of Quer for hypoxia-induced cells compared to NC (Figure 4E). The expressions of cleaved-caspase-7/3/9 were enhanced in miR-122 overexpressed with Quer treatment in hypoxia-induced cells compared to NC but were reduced in miR-122-silenced cells (Figure 4F). These data suggest that Quer protected PC-12 against hypoxia injury may be through down-regulating miR-122. 

***AMPK and Wnt/β-catenin signaling pathways participated in the function of Quer***

Hypoxia treatment induced decreased expression of p-AMPK in PC-12 cells (*P*<0.05). However, compared to hypoxia treatment, p-AMPK expression was significantly increased in PC-12 cells suffered by hypoxia and treated with Quer compared to control (*P*<0.01). However, miR-122 overexpression significantly repressed p-AMPK expression (*P*<0.01) by treatment with Quer in hypoxia-induced PC cells compared to NC, while silence of miR-122 had no significant effect on p-AMPK expression (Figure 5A). Similarly, hypoxia treatment induced significantly decreased expression of β-catenin in PC-12 cells (*P*< 0.01). Then, its expression was significantly elevated by Quer treatment (*P*<0.01). High level of miR-122 impaired the effect of Quer on β-catenin expression with diminishing β-catenin expression compared to NC (*P*<0.01). However, β-catenin expression was further increased by silence of miR-122 in Quer-treated hypoxia-induced PC-12 cells (*P*<0.05) (Figure 5B). These results indicated that Quer protected PC-12 cells against hypoxia injury possibly by activating AMPK and Wnt/β-catenin signaling pathways via down-regulation of miR-122. 

## Discussion

The role of Quer in modulation of hypoxia injury in PC-12 cells was investigated and the underlying mechanism of Quer was explored in this study. According to our data, Quer ameliorated hypoxia-induced injury in PC-12 cells by down-regulating miR-122, during the process in which AMPK and Wnt/β-catenin signaling pathways were activated.

Neonatal hypoxic brain injury is one of primary reasons for mortality and morbidity in infancy and childhood; however, there is no therapeutic strategy for the moment (23). Although many trials have concentrated on novel therapies, very few studies have had limited clinical benefits because of lacking understanding of the discrete cellular and molecular factors and signaling pathways participating in hypoxic brain injury mechanisms (23, 24). 

In our study, hypoxia-induced PC-12 cells exhibited decreased viability, depressed migration, and increased apoptosis. Meanwhile, expression of Cyclin D1 was down-regulated, while p16 was up-regulated by hypoxia. Cyclin D1 and p16 are molecules with pivotal roles in cell cycle control and the development of diverse human cancers (25). Aberrant Cyclin D1 and p16 expression was related to various diseases (26-28). Cyclin D1 serves as a key sensor and integrator of extracellular signals of cells in G0 and early G1 phases (29), mediating its function through binding with cyclin-dependent kinase (CDK) 4/6 (30), leading to cell cycle progression, whereas p16 inhibits CDK4, preventing cell cycle progression. In addition, hypoxia and the regulation of cell cycle are closely related (31). A previous study revealed that hypoxia stimulated p16 expression (32), while dramatically down-regulated Cyclin D1 in PC-12 cells (33). Our results were consistent with these reports. Taken together, hypoxia successfully induced cell injury in PC-12 cells. 

Furthermore, we performed our study to investigate the effects of Quer on hypoxia-induced PC-12 cells. Results demonstrated that Quer attenuated hypoxia-induced injury by affecting on cell viability, migration and apoptosis. Previous studies revealed that intravenous nanosomes of Quer improved brain function and hemodynamic instability after severe hypoxia in newborn piglets (34). In addition, Quer ameliorated hypobaric hypoxia-induced memory impairment through mitochondrial and neuron function adaptation (35). Furthermore, Quer can be used as a novel therapy for inflammatory hypoxic stress in rats (36). Our results, consistent with the results in the past, showed that Quer alleviated hypoxia-induced injury. 

miRNAs are involved in various biological functions by acting as inhibitors of gene expression. miR-122 was found to be a tumor suppressor that regulated metastasis and progression of hepatocellular carcinoma (37) and gastrointestinal cancer (38). A few studies evaluated the cytoprotective effect of miR-122 silence in the *in vitro *model of hypoxic injuries. For example, down-regulating miR-122 attenuated hypoxia/reoxygenation-caused myocardial cell apoptosis (39); the excessive expression of miR-122 increased hypoxia-induced proliferation and migration loss of H9c2 cells, and promoted cell apoptosis, but oppositely, repressed expression of miR-122 improved cell viability and inhibited cell apoptosis (40). miR-122 also played a crucial role in protection of hepatocytes against hypoxia injury (41). However, there has been no study assessing the role of miR-122 in hypoxia injury in PC-12 cells at present. In our study, we found that Quer treatment down-regulated miR-122 expression in hypoxia-induced PC-12 cells. miR-122 overexpression attenuated the promoting effects of Quer on growth and migration of PC-12 cells, which was consistent with previous studies. 

AMPK pathway was involved in neonatal hypoxic–ischemic brain injury in mice (42). In addition, Quer function was regulated by AMPK signaling pathway (43). For example, Quer ameliorated glucose uptake against type 2 diabetes through AMPK pathway in skeletal muscle cells (44). In our study, we found that Quer activated AMPK signaling pathway through down-regulation of miR-122 in hypoxia-induced PC-12 cells. This was consistent with the results of Rousset* et al. *demonstrating that reduced AMPK activity promoted injury (42). 

Wnt/βcatenin signaling pathway was observed to play an important role in neonatal brain injury (45). Our findings showed that Quer activated Wnt/β-catenin signaling pathways by down-regulation of miR-122, which was consistent with the result from Xu *et al.* Overexpression of miR-122 down-regulated expression of β-catenin, which inactivated Wnt/β-catenin signaling pathway (46), while opposite results were reported by Nallar *et al. *indicating that hypoxia induces the activation of the Wnt/β-catenin signaling (47).

## Conclusion

Overall, Quer displayed neuroprotective effect on PC-12 cells exposed to oxygen deprivation. Quer attenuated hypoxia injury in PC-12 cells by enhancing growth and migration. Activations of AMPK and Wnt/β-catenin signaling pathways modulated by miR-122 down-regulation contributed to the neuroprotective function of Quer. This study might open a new horizon for amelioration of neuronal cell damage in cerebral anoxia. 
